# Characterization of miRNA profiles in the mammary tissue of dairy cattle in response to heat stress

**DOI:** 10.1186/s12864-018-5298-1

**Published:** 2018-12-28

**Authors:** Qiuling Li, Chunhong Yang, Juan Du, Baogui Zhang, Ying He, Qimeng Hu, Meiru Li, Yiming Zhang, Changfa Wang, Jifeng Zhong

**Affiliations:** 1 Edible and Medicinal Fungi Research and Development Center, College of Life Sciences, Langfang Normal University, Langfang, 065000 People’s Republic of China; 2 Dairy Cattle Research Center, Shandong, Academy of Agricultural Science, Jinan, 250100 People’s Republic of China; 3 Yongqing Animal Husbandry and Veterinary Bureau, Yongqing, 065600 People’s Republic of China; 4 Dachang County Animal Health Supervision Institute, Dachang, 065300 People’s Republic of China

**Keywords:** Dairy cattle, Heat stress, Mammary gland, MicroRNA

## Abstract

**Background:**

MicroRNAs (miRNAs) are a class of small noncoding RNAs that play important roles in the regulation of gene expression. However, the role of miRNAs in bovine mammary gland responses to heat stress is not well understood.

**Results:**

In the present study, we performed a deep RNA sequencing analysis to identify miRNAs associated with the heat stress potential of the bovine mammary gland. We identified 27 miRNAs that were differentially expressed significantly between the mammary tissue of Holstein cattle heat stress and normal conditions. Twenty miRNAs had higher expression in the mammary tissue of heat-stressed Holstein cattle. The seven highest differentially expressed candidate miRNAs (bta-miR-21-5p, bta-miR-99a-5p, bta-miR-146b, bta-miR-145, bta-miR-2285 t, bta-miR-133a, and bta-miR-29c) identified by deep RNA sequencing were additionally evaluated by stem-loop qPCR. Enrichment analyses for targeted genes revealed that the major differences between miRNAs expression in the mammary gland of heat-stressed versus control were associated with the regulation of Wnt, TGF-β, MAPK, Notch, and JAK-STAT.

**Conclusions:**

These data indicated that the differentially expressed miRNAs identified in this study may act as dominant regulators during heat stress. We might reduce heat stress damage of Holstein cows by up-regulating or down-regulating these differentially expressed miRNAs.

**Electronic supplementary material:**

The online version of this article (10.1186/s12864-018-5298-1) contains supplementary material, which is available to authorized users.

## Background

Stress can be defined as an external condition that produces a “strain” in a biological system [1]. The environmental stress may be measured by changes in body temperature, metabolic rate, or productivity. Heat stress negatively impacts all features of dairy cattle production including milk composition and mammary gland pathogens. It substantially influences a cow’s growth and development [2]. Reduction in reproductive performance of lactating cows during summer is associated with decreased thermoregulatory competence [3]. Heat stress causes cow metabolic disorders and a reduction in milk production [4, 5], and it also decreases immunity and increases susceptibility to mastitis, endometritis disease and even death in severe cases [6–8]. Diminished milk yield and reproductive losses during summer months seriously affect the economic potential of the dairy industry. In addition, global warming may boost the occurrence of heat stress [9]. Thus, for the dairy industry, heat stress has been a bottleneck which limits the efficiency of the dairy supply throughout the year.

The heat stress response is a complex molecular process that involves the transcriptional and post-transcriptional regulation of stress-related genes. Acute environmental change initiates the heat stress response at the cellular level. Changes in gene expression are associated with a reaction to an environmental stressor as well as changes across a variety of organs and tissues associated with the acclimation response. Functional genomics establishes a verifiable link between gene expression and phenotype. Endogenous noncoding small RNAs known as microRNAs (miRNAs) are increasingly being recognized as important modulators of gene expression at the post-transcriptional level and have been shown to be involved in diverse biological processes such as differentiation, development, apoptosis, and viral infection. RNA-Seq, in particular, allows a global analysis of gene expression responses to environmental change.

Some miRNAs have been shown to be involved in plant stress responses by down-regulating the respective target genes encoding regulatory and functional proteins [10]. Differential expression of miRNAs is also associated with thermal stress in cattle. Down-regulation of miR-181a can reduce heat stress damage in peripheral blood mononuclear cells (PBMCs) of Holstein cows [11]. miRNA profiles in bovine mammary tissue infected with *Staphylococcus* [12, 13] and miRNA profiles in serum or PBMC cell of heat-stressed cattle have been studied [14–16]; however, miRNA profiles in bovine mammary gland of stressed lactating cattle have not been compared to normal lactating cattle. Therefore, it was our objective to profile miRNA expression under heat stress using bovine mammary glands. To investigate the role of miRNA in the heat stress response, miRNA expression in bovine mammary glands were characterized by next-generation sequencing during summer and spring with and without a heat stress challenge.

## Methods

### Tissue collection

Samples were collected from eight mammary gland tissues of the four lactating Chinese Holstein cows from Yucheng dairy farm, China. The samples were collected in two different environmental seasons, viz. spring and summer, with the temperature ranges between 15 and 20 °C (March; designated as non-heat stressed, NHS) and 30–38 °C (July; designated as heat stressed, HS), respectively. The temperature humidity index (THI) was used as a heat stress indicator. The temperature probing procedure has been described in detail elsewhere [17]. Dry-bulb and wet-bulb temperatures were recorded using a dry and wet bulb thermometer consisting of two thermometers, a dry bulb thermometer and a wet bulb thermometer. THI was calculated as THI = 0.72 (Td + Tw) + 40.6 where Td is the dry-bulb temperature and Tw is the wet-bulb temperature. Stress response of the animals was characterized by recording physiological parameters. The rectal temperature (RT) and heat tolerance coefficient (HTC) were measured for both NHS and HS groups according to a method previously described, HTC = 100–10 (RT-38.3) [18]. Four cows were designated as biological replicates for NHS and HS. In each case, one complete mammary gland was removed after excision of the intramammary lymph node. The samples were frozen in liquid nitrogen until RNA extraction.

### Total RNA extraction

Total RNA from the mammary gland was isolated using Trizol Reagent (Life Technologies, USA) according to manufacturer’s instructions. RQ1 DNase (Promega, USA) was used to treat the total RNA to digest the DNA. The concentration and purity of the extracted RNA were determined with a NanoDrop 1000 Spectrophotometer (NanoDrop Technologies, USA). The quality of RNA was assessed through the Agilent 2100 Bioanalyzer (Agilent Technologies, USA). All eight RNA samples had an RNA integrity number (RIN) of ≥8 and were stored at − 80 °C for further analysis.

### Small RNA library construction

Equal amounts of total RNA (330 ng) from each mammary gland sample of both NHS and HS groups were used to construct a miRNA library through a TruSeq Small RNA Sample Preparation kit (Illumina, USA) following the manufacturer’s protocol. PCR amplification was performed including 13 cycles. Eight small RNA libraries were constructed in equal amounts for gel purification. Quality and quantity of purified small RNA were estimated using an Agilent 2100 Bioanalyzer (Agilent Technologies, USA). Sequencing was carried out on a HiSeq-2000 system (Illumina, USA). Real-Time Analysis (RTA) and base calling were performed by HiSeq Control Software Version 1.4.8 (Illumina, USA).

### Small RNA sequence analysis

Low-quality reads were removed from the raw data. After trimming the 3′ adaptor sequence, small RNA reads with the length of 18–30 nt from all libraries were extracted using miRDeep2 (version 2.0.0.5) with the default parameters to identify known and novel miRNAs [19]. Each library was processed separately. Subsequently, all reads were mapped to the bovine genome with Bowtie [20]. The reads that mapped to bovine tRNAs, rRNAs, and snoRNAs in the Rfam RNA family database [21] were discarded. Small RNAs that only mapped to genomic repeat loci were removed. Novel miRNA and precursors were identified by the core module miRDeep2.pl. Datasets of novel miRNA and precursors were created through adding miRNA predicted with a miRDeep2 score > 0 to known miRNA. The expression of detected miRNAs for each library was estimated by the Quantifier module of miRDeep2 [19]. To investigate the regulation of miRNAs in mammary gland in response to heat stress, miRNA expression under heat stress (summer) were compared to expression in controls (spring) using DEseq [22].

### Target gene prediction and pathway analysis

The target genes of differentially expressed miRNAs were predicted through TargetScan (http://www.targetscan.org) and miRanda (http://www.microrna.org/microrna/home.do) according to a previous study [23]. To comprehensively describe the properties of genes and gene products, we executed gene ontology (GO) annotation and enrichment analysis from three ontologies: molecular function, cellular component and biological process. Functional analysis of predicted gene targets was performed using the Database for Annotation, Visualization and Integrated Discovery (DAVID version 6.7, http://david.abcc.ncifcrf.gov/) for pathway analysis.

### Stem-loop quantitative PCR and data analysis

Seven differentially expressed miRNAs (bta-miR-21-5, bta-miR-99a-5p, bta-miR-146b, bta-miR-145, bta-miR-2285 t, bta-miR-133a, and bta-miR-29c) were validated through a standardized and reliable stem-loop qRT-PCR procedure. The RT reaction mixture contained a 4 μg aliquot of total RNA and a mixture of 1 μL of each RT primers (10 μM) for all of the mature miRNAs and U6, which was chosen as a reference control (Additional file 1), 10 μL 2 × reaction mix, 1.5 μL RT enzyme mix (Sangon, China). The mixture was incubated at 16 °C for 30 min, 37 °C for 30 min, 85 °C for 5 min, subsequently, frozen on ice for at least 5 min. SYBR® Green Realtime PCR Master Mix (Sangon, China) was used to detect miRNA expression by a Bio-Rad IQ5 System (USA). Briefly, cDNAs were diluted 10 times and a 1 μL diluted sample was used as a template in a 20 μL PCR reaction, which contained 10 μL 2 × SYBR Green Realtime PCR Master Mix, 0.25 μM of a miRNA-specific forward primer and universal reverse primer, respectively. The quantitative PCR was conducted in triplicate for 1 min at 95 °C, followed by 40 cycles of 15 s at 95 °C and 30 s at 60 °C. For each PCR, dissociation curve analysis was carried out to discriminate specific products from primer dimers. The fold changes of miRNA in different samples were calculated by ΔCt method.

The relative expression of target miRNA was calculated by the following formula: ΔCt (target miRNA) = Ct (target miRNA)–Ct (internal reference). U6 was used as an internal reference. The NHS group at spring was selected as a calibrator for the relative quantification. The relative expression of miRNA normalized to internal control and relative to the calibrator was calculated as follows: Relative expression of target miRNA = 2^-ΔΔCt^, where ΔΔCt = ΔCt (target miRNA, sample) –ΔCt (target miRNA, calibrator). The difference between the two groups was compared using T-test (SAS version 9.2, 2008). Statistical difference was at *P* < 0.05.

## Results

### Characterization of the stress response

Rectal temperature and HTC at two different environmental temperature ranges are presented in Table 1. A THI of above 72 and a rectal temperature of above 39 °C were regarded as an indication of heat stress. The physiological parameters of HS were significantly different from the NHS group of cows (*P* < 0.05). The average rectal temperature of HS group was higher, and a significant decrease of HTC as compared with the control. Rectal temperature and HTC data showed that the cattle were under heat stress.

**Table 1:** Physiological parameters recorded during different environmental temperature

Group	Environmental temperature ranges (°C)	THI	Rectal temperature (°C)	HTC
NHS	15–20	65.8	38.35 ± 0.23^a^	99.50 ± 2.32 ^a^
HS	30–38	83.8	39.43 ± 0.24^b^	88.75 ± 2.44 ^b^

Different superscript letters indicate significant differences at *P* < 0.05

*NHS* non-heat stressed group, *HS* heat stressed group, *THI* temperature humidity index, *HTC* heat tolerance coefficient

### miRNA sequencing

**Fig. 1:**
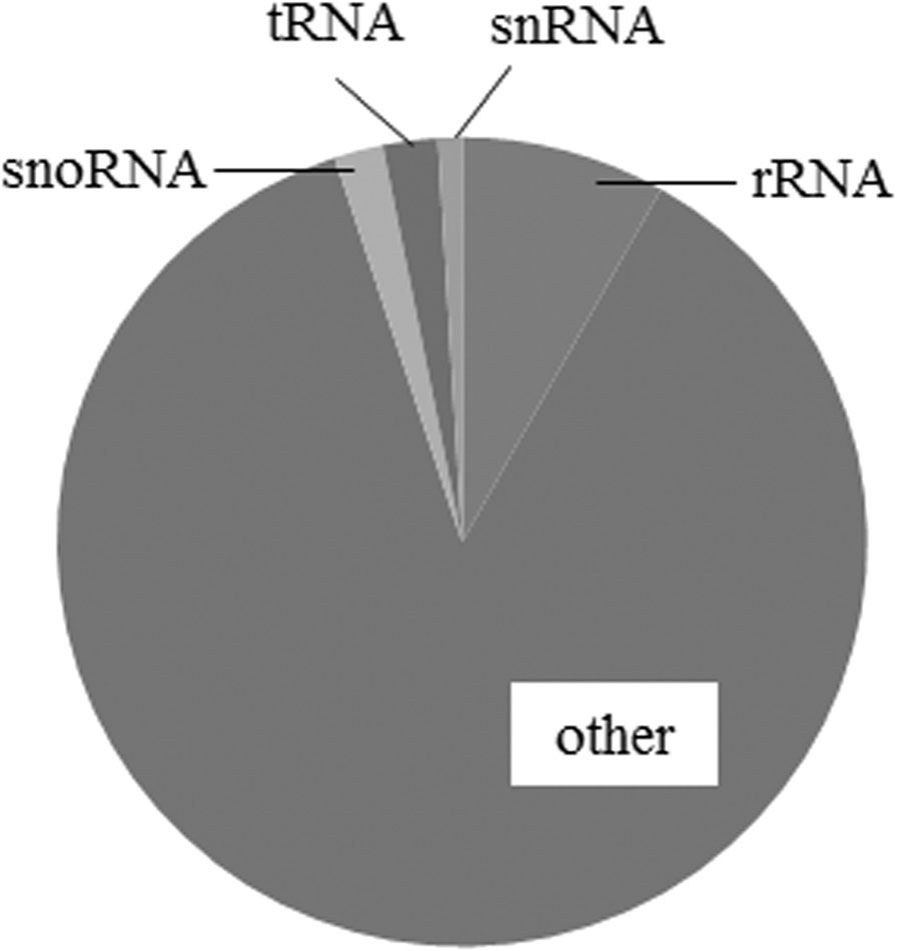
A pie graph showed relative abundance of different classes of small RNAs. Of the 18 to 30 nucleotide sRNA fraction, four out of five (86.72%) were identified as other noncoding sRNA, while only a small number (13.28%) aligned to rRNAs, tRNAs, snRNAs and snoRNAs in bovine

**Fig. 2:**
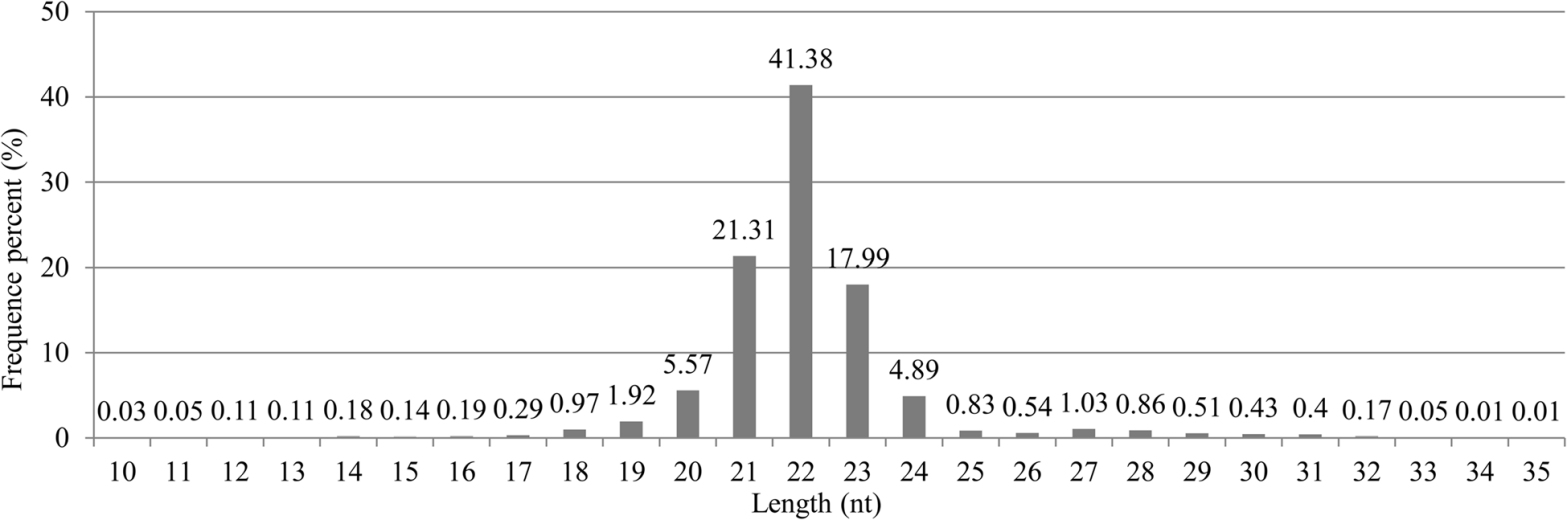
Frequency distribution of detected small RNAs (18–30 nt)based on all reads. The major reads from small RNAs were 20 to 24 nucleotides in length (accounting for 91.14% of total number). Dominant reads of small RNAs were 22 nucleotides in length (41.38%), followed by 21 and 23 nucleotides, and lastly 20 and 24 nucleotides

Eight small RNA libraries were constructed and sequenced. A total of 20,818,666 high-quality reads were generated. Among them, 19,859,218 sequences ranging from 18 to 30 nucleotides were obtained after adaptor trimming, accounting for 95.39% of all small RNA (sRNA) sequences. A summary of the data is provided in Table 2. Alignment with miRBase (Release 21) indicated that miRNAs were highly enriched in all libraries. To assess the efficiency of high-throughput sequencing for sRNA detection, all sequence reads were annotated and classified by alignment with Rfam databases. Of the 18 to 30 nucleotide sRNA fraction, four out of five (86.72%) were identified as other noncoding sRNA, while only a small number (13.28%) aligned to rRNAs, tRNAs, snRNAs and snoRNAs in bovine (Fig. 1). The major reads from sRNAs were 20 to 24 nucleotides in length (accounting for 91.14% of total number). Dominant reads of sRNAs were 22 nucleotides in length (41.38%), followed by 21 and 23 nucleotides, and lastly 20 and 24 nucleotides (Fig. 2).

**Table 2:** Statistics of miRNA-seq data for control and heat stressed dairy cow mammary gland

Sample	Total reads	High quality reads	3′ adapter null	Insert null	5′ adapter contaminants	Clean reads
1	23,740,518	23,684,283	352,584	149,789	10,795	22,775,357
2	18,179,219	18,138,345	266,264	78,422	7726	17,380,420
3	21,734,611	21,684,303	324,037	64,043	5398	21,042,828
4	18,781,285	18,738,780	273,308	94,522	14,550	17,894,570
5	21,752,063	21,703,699	338,405	188,847	9943	20,759,707
6	18,485,467	18,443,383	345,756	174,824	15,332	17,214,481
7	20,690,730	20,637,198	236,984	171,114	12,944	19,673,600
8	23,575,000	23,519,333	525,390	263,643	11,551	22,132,782
Sum	166,938,893	166,549,324	2,662,728	1,185,204	88,239	158,873,745
Average	20,867,362	20,818,666	332,841	148,151	11,030	19,859,218

Total reads, total sequenced reads; High quality reads, number of high quality reads with no N, no more than 4 bases whose quality score is lower than 10 and no more than 6 bases whose quality score is lower than 13; 3′ adapter null, number of reads with no 3’adaptor; Insert null, number of reads with no insertion; 5′ adapter contaminants, number of 5′ contaminants; Clean reads, number of clean reads after adaptors and contaminants are removed which are used in the following analysis

We identified 483 known bovine miRNAs (counts per million, CPM > 10 in at least one library). Among them, eight highly expressed miRNAs accounted for 35.41% of the total reads of identified known miRNAs (Table 3). The four highest expressed miRNAs were bta-miR-21-5p, bta-let-7a-5p, bta-miR-26a, and bta-miR-148a, accounting for 9.15, 5.55, 5.46, and 4.45% of total known miRNA reads, respectively.

**Table 3:** Top 8 expressed miRNAs in bovine mammary gland with or without heat stress

miRNA ID	Total no. of reads	Ratio		
		All samples	Control group	Heat stressed group
bta-miR-21-5p	15,246,710	9.15%	12.07%	6.31%
bta-miR-26a	9,094,751	5.46%	5.98%	4.95%
bta-let-7a-5p	9,246,889	5.55%	5.56%	5.55%
bta-miR-99a-5p	3,910,569	2.35%	3.10%	1.62%
bta-miR-143	5,040,976	3.55%	3.56%	3.55%
bta-let-7b	5,377,139	3.23%	3.63%	2.84%
bta-miR-148a	7,414,411	4.45%	3.70%	5.19%
bta-let-7f	3,643,338	2.19%	2.23%	2.15%

Ratio refers to the total number of reads of a miRNA as compared to all reads of known bovine miRNAs (miRBase Release 21) detected in all, control, and heat stressed samples, respectively

### Identification of novel miRNAs and miRNA candidates

To determine whether these small RNA sequences are genuine bovine miRNAs, we scanned the bovine genome for hairpin structures comprising the candidate miRNA with miRDeep2 software (version 2.0.0.5), which can be used to identify both known and novel miRNAs from deep sequenced sRNA libraries. In total, 483 loci possessed the typical stem-loop structures matching the known miRNA hairpins (808 miRNA, miRbase 21) in the mammary gland. A total of 139 novel miRNA hairpins were identified (Additional file 2).

**Fig. 3:**
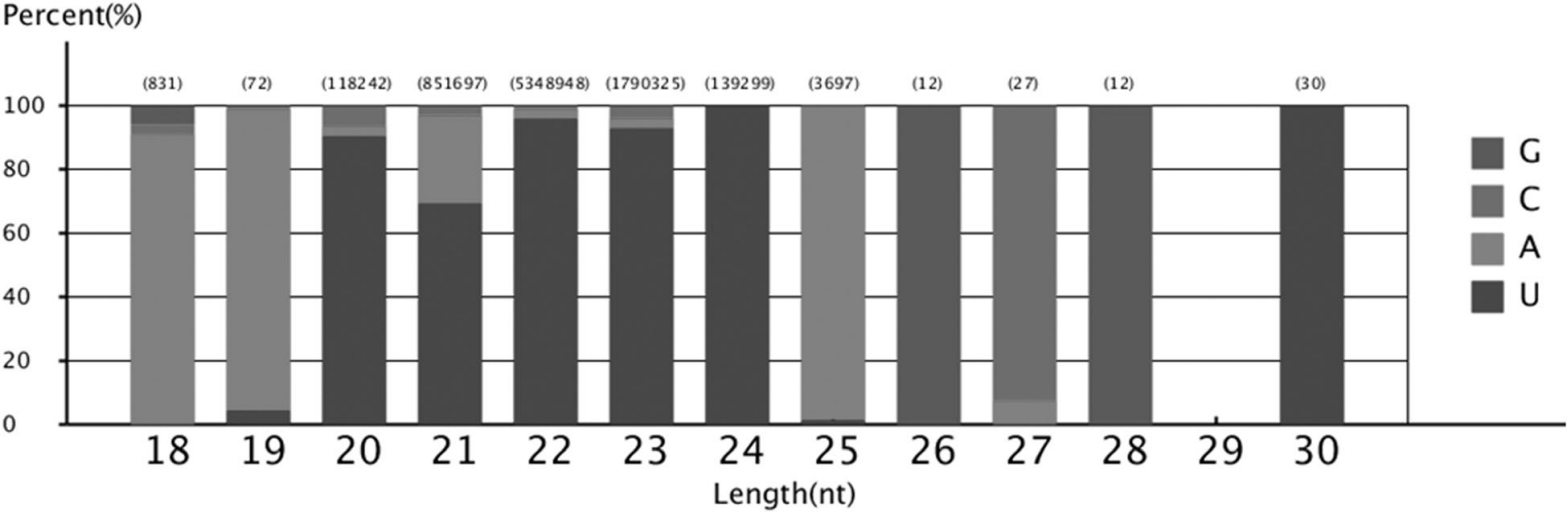
First nucleotides bias of 18–30 nt miRNAs candidates. Uridine (U) was the most common at the 5′ end of the 20–24 nt miRNAs in the mammary gland

**Fig. 4:**
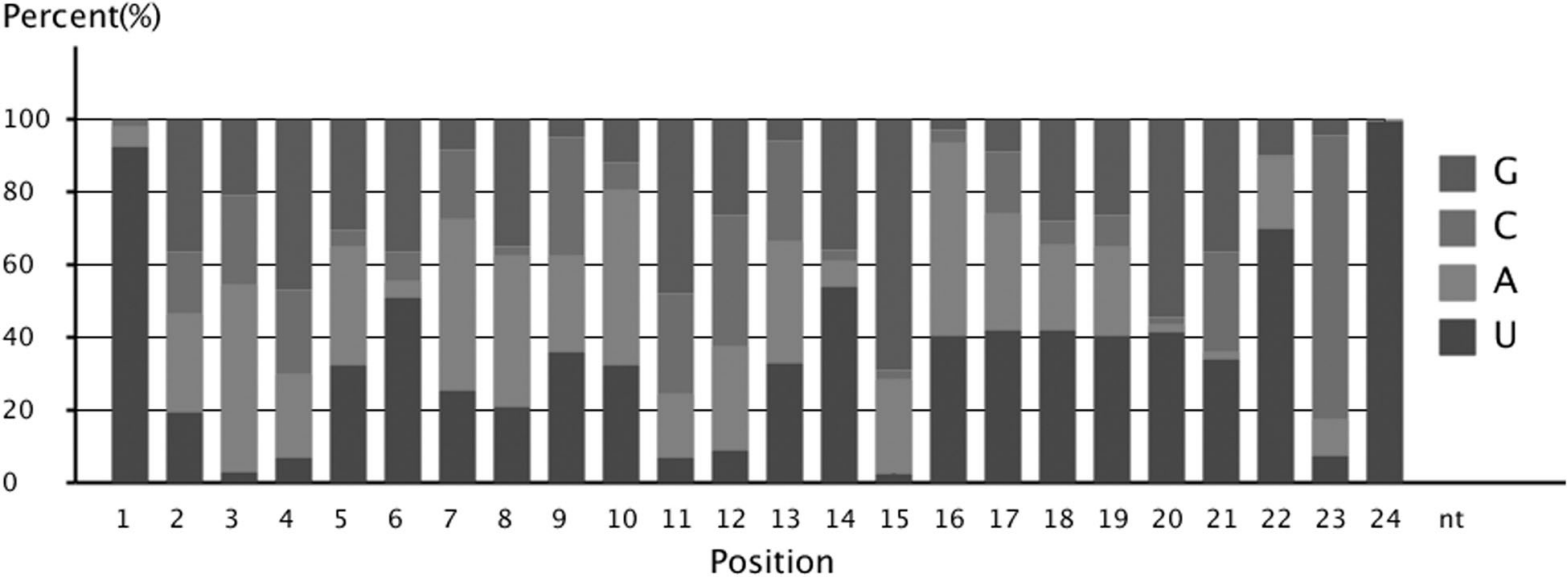
Nucleotides bias at each position of miRNA. The U nucleotide was the dominant first nucleotide, while the G content was very low at position 1. The U nucleotide had a low frequency in the 2th, 3th, and 4th positions

Analyses of the first nucleotide bias of the 18–30 nt miRNAs candidates revealed that uridine (U) was the most common at the 5′ end of the 20–24 nt miRNAs in the mammary gland (Fig. 3). Moreover, miRNA nucleotide bias at each position also showed that U was the dominant first nucleotide (Fig. 4).

### Effects of heat stress on miRNA expression in mammary gland

**Fig. 5:**
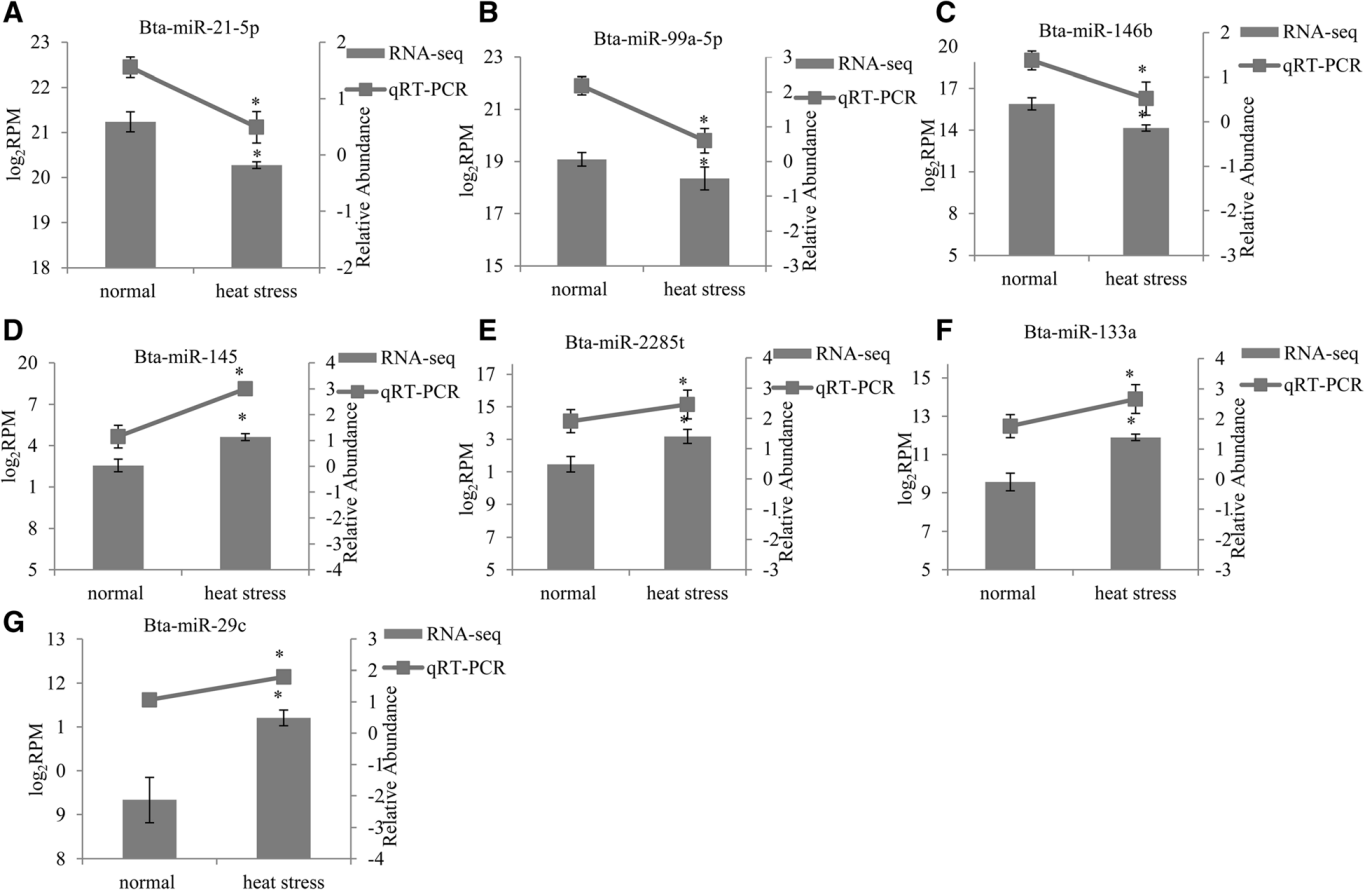
Expression of differentially expressed miRNAs between heat stressed and normal conditions detected by qRT-PCR and RNA-seq. **a** Expression of bta-miR-21-5p; **b** Expression of bta-miR-99a-5p; **c** Expression of bta-miR-146b; **d** Expression of bta-miR-145; **e** Expression of bta-miR-2285 t; **f** Expression of bta-miR-133a; **g** Expression of bta-miR-29c. Lines on the top represented miRNA expression from qRT-PCR and values were showed on the right vertical axis as relative abundance. Bars on the bottom represented miRNA expression from RNA-seq and values were showed on the left vertical axis as log_2_RPM (normalized reads number). * on the top of lines or bars indicated significant difference (*P* < 0.05 or FDR < 0.05) between heat stressed and normal condition. Data were presented as Mean ± Standard Error

Given that miRNAs play important roles in many biological processes, we speculated that the expression of miRNAs might be regulated in the mammary glands of Holstein cattle under heat stress. The global expression of miRNAs under normal condition and heat-stressed condition were profiled and the correlations between libraries were performed with the normalized counts of all detected miRNAs. Only a small number of miRNAs were significantly regulated in mammary glands of Holstein cattle under heat stress. DEseq, an R/Bioconductor package [22] method was used to analyze differentially expressed miRNAs between different conditions based on sequence counts. We observed that 24 miRNAs were differentially expressed (*P* < 0.05) in the heat stress condition when compared to the control (Additional file 3). The highest differentially expressed miRNAs (bta-miR-21-5p, bta-miR-99a-5p, bta-miR-146b, bta-miR-145, bta-miR-2285 t, bta-miR-133a, and bta-miR-29c) are shown in Fig. 5. The expression of bta-miR-145, bta-miR-2285 t, bta-miR-133a, and bta-miR-29c was increased and reached a 3.50, 3.24, 4.30, and 4.03-fold increase (*P* < 0.05), respectively. Conversely, the expression of bta-miR-21-5p, bta-miR-99a-5p, and bta-miR-146b were decreased under heat stress condition (*P* < 0.05). Furthermore, we also verified the expression of these miRNAs using qRT-PCR (*P* < 0.05) and the expression pattern was consistent with sequencing results (Additional file 4).

### Biological function enrichment analysis of predicted target genes of differentially expressed miRNAs in mammary glands of Holstein cattle under heat stress

**Fig. 6:**
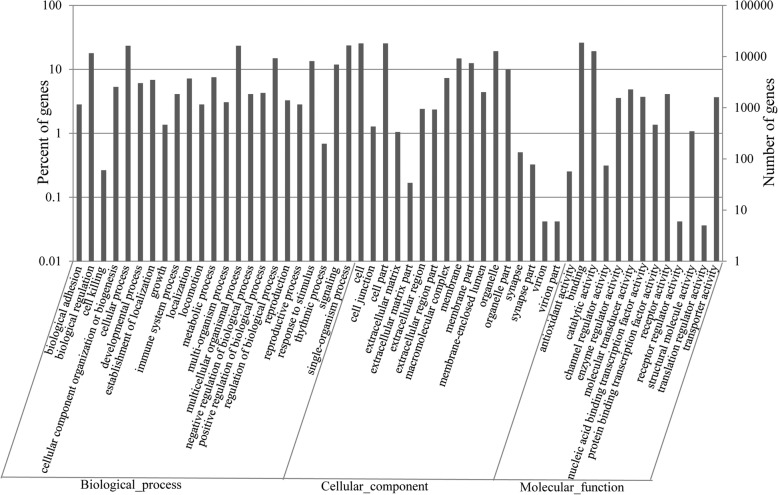
GO analysis of predicted target genes of differentially expressed miRNAs during heat stressed challenge of bovine mammary gland. X axis: GO classification (biological process, cellular component, and molecular function). Y axis: Left, percentage of genes; Right, number of genes in this term

Target gene prediction of 24 miRNAs differentially regulated by heat stress indicates that about 26,824 genes that were enriched in 59 GO terms may be regulated by these miRNAs (Fig. 6). GO analysis showed that the predicted targets of differentially expressed miRNAs were significantly enriched (*P* < 0.05) in different functional groups, namely, biological regulation, cellular process, metabolic process, multicellular organismal process, regulation of biological process, response to stimulus and single-organism process (Fig. 6, Table 4). One of the GO terms included more than 2000 responses to stimulus-related genes. Interestingly, several KEGG (Kyoto encyclopedia of genes and genomes) pathways were significantly enriched (*P* < 0.05) by target genes of the 24 differentially expressed miRNAs (Table 5). Notably, pathways of the RNA degradation, mTOR signaling pathway, immune system and pathways in human diseases, especially cancer were significantly enriched by target genes. This indicated that the differentially expressed miRNAs identified in this study might act as a dominant regulator during heat stress.

**Table 4:** GO functional analysis of the differentially expressed miRNA potential targets

GO term	Count	Frequency (%)
Biological regulation	7893	34.05
Cellular process	15,589	67.25
Metabolic process	12,074	52.09
Regulation of biological process	7242	31.24
Response to stimulus	6626	28.59
Single-organism process	13,247	57.15
Cell	16,922	73.01
Cell part	16,922	73.01
Membrane	8580	37.02
Membrane part	6913	29.82
Organelle	11,950	51.56
Binding	15,556	67.11
Catalytic activity	8773	37.85

**Table 5:** KEGG pathway annotation of the miRNA potential targets

Pathway ID	Pathway name	Target genes with pathway annotation (51813)	All genes of the *Bos taurus* with pathway annotation (55072)	*P* value
ko04510	Focal adhesion	1593 (3.07%)	1628 (2.96%)	9.23E −14
ko04810	Regulation of actin cytoskeleton	2166 (4.18%)	2218 (4.03%)	2.16E −16
ko04530	Tight junction	2000 (3.86%)	2027 (3.68%)	3.23E −26
ko00500	Starch and sucrose metabolism	221 (0.43%)	222 (0.4%)	1.92E −05
ko01110	Biosynthesis of secondary metabolites	1043 (2.01%)	1084 (1.97%)	9.24E −04
ko04670	Leukocyte transendothelial migration	858 (1.66%)	875 (1.59%)	7.30E −09
ko04350	TGF-beta signaling pathway	367 (0.71%)	377 (0.68%)	2.31E-03
ko04310	Wnt signaling pathway	855 (1.65%)	892 (1.62%)	1.14E-02
ko04330	Notch signaling pathway	299 (0.58%)	310 (0.56%)	4.19E-02
ko04150	mTOR signaling pathway	315 (0.61%)	326 (0.59%)	2.64E-02
ko04011	MAPK signaling pathway - yeast	118 (0.23%)	120 (0.22%)	2.43E-02
ko04630	JAK-STAT signaling pathway	633 (1.22%)	656 (1.19%)	3.33E-03
ko04012	ErbB signaling pathway	505 (0.97%)	519 (0.94%)	4.38E-04

Target genes with pathway annotation, The number of target genes which are associated with the pathway (ratio). All genes of the bos taurus with pathway annotation, The number of all reference genes which are associated with the pathway (ratio)

## Discussion

Heat stress triggers a dramatic and complex program of altered gene expression in mammary glands similar to patterns investigated in other cell types exposed to thermal stress. As reported by Sonna et al. [24], these changes include inhibition of DNA synthesis, RNA transcription, and translation, disruption of cytoskeletal components, and alterations in metabolism. In the present study, thermal stress induced changes in the miRNA expression in dairy cattle mammary glands. Although the miRNA expression profiles in dairy cattle have been studied, there is still a limited understanding of their role in heat-stressed bovine mammary glands and the influence of this on dairy potential. In this study, 483 known miRNAs were detected in dairy cattle mammary glands by RNA-Seq.

Several studies have uncovered the roles of the highly abundant miRNAs in mammary glands. For example, two of the eight miRNAs with the highest expression in the dairy cattle mammary gland in this study, bta-miR-21-5p, the other arm of bta-miR-21, and bta-miR-143, were shown to promote adipogenesis [25, 26], which accounts for 12.70% of the miRNAs expressed in the dairy cattle mammary gland. The expression of miR-21-5p was strongly induced at 7 d postpartum compared with the dry period suggesting that it might promote mammary cell proliferation during early lactation [27]. MiR-21 decreases the expression of Tgfbr2 by targeting TGF b receptor II (Tgfbr2) and eventually enhances adipogenic differentiation [26]. Moreover, miR-21 is associated with thermal stress in Frieswal crossbred dairy cattle [15]. Our data showed that the expression of bta-miR-21-5p was lower in heat stressed group than that in control, which is consistent with bta-miR-21 expression in PBMC of Sahiwal cows [16]. The up-regulation of miR-143 decreased the expression of pleiotrophin and increased some adipocyte-important genes, enhancing the rate of adipocyte differentiation at early stages of adipogenesis [25]. Decreased expression of miR-143 by its antisense sequence suppressed differentiation of preadipocytes through repressing ERK5, suggesting this miRNA may play a key role in adipocyte differentiation [28].

One of the other eight most abundant miRNAs in this study is miR-148a, orthologs of miR-148. MiR-148 was highly abundant in the lactating mammary gland of mouse and goat [29, 30]. MiR-148a and miR-26a show the similar expression patterns during the lactation period in cow milk [31]. Accumulating evidence indicates that miR-148a induce cell proliferation and differentiation [32, 33]. MiR-148a promotes adipogenesis by repressing Wnt signaling [34]. In addition, the expression of bta-miR-145, bta-miR-133a, and bta-miR-29c was increased in the heat-stressed group. MiR-145 is associated with cow mastitis caused by *Staphylococcus aureus* [12]. Moreover, the expression of miR-145 during differentiation can regulate the insulin receptor substrate 1 to inhibit adipogenesis [35]. Inhibition of miR-133 led to the expression of GLUT4 and insulin-mediated glucose uptake attenuation in cardiomyocytes [36]. Overexpression of miR-29a-c reduced the protein levels of PGC-1a and G6 Pase in primary hepatocytes and mouse livers [37]. These researches indicate that the highly expressed miRNAs may be related to the mammary gland biology, milk synthesis, and lactation process.

Bta-miR-2285 t was significantly increased in the mammary tissue of dairy cattle (Holstein-Friesian) compared with beef (Limousin) postpubertal heifers [38]. However, its role in the mammary gland remains unclear. The expression of bta-miR-2285 t and bta-miR-146b was decreased in the heat-stressed group compared with the control. Similarly, miR-146b has a lower expression in the serum of heat-stressed Holstein cows [14]. It has been suggested that miR-146b can modulate Sirtuin1, suppressing the negative regulators of adipogenesis, and eventually promoting adipogenesis [39]. Up-regulation of miR-146b is found during pregnancy, especially in the luminal progenitors compared to the basal/stem cells, suggesting it is involved in the differentiation of mammary epithelial cells [40]. Furthermore, the expression of miR-146b was upregulated in the luminal progenitors in pregnant mice, which indicates that miR-146b is involved in the differentiation of the mammary stem cells [40].

Interestingly, we found that the expression of bta-miR-21-5p and bta-miR-146b tended to decrease, and bta-miR-145 tended to increase in the heat-stressed group compared with control group. These results are consistent with the phenomenon that, acute heat stress affects the lipolysis and the rate-limiting enzyme of lipogenesis in bovine adipocytes [41]. Considering that adipocytes in the mammary gland can regulate the growth and biological function of the mammary epithelium [42], we speculated that these miRNAs might have a role in the regulation of milk fat synthesis.

A total of 139 novel miRNA hairpins were detected. High-throughput sequencing and bioinformatics analysis have become the main methods to identify the potentially novel miRNAs since 2008 [43]. Given the number of known miRNAs (808 miRNAs) in the miRBase (Release 21), our data may enrich the miRNA resources in bovine.

To further understand and provide some molecular insight into the physiological functions and biological processes involving these miRNAs in heat-stressed mammary glands, target genes were predicted based on miRNA/mRNA interactions. The predicted target genes were classified using KEGG function annotations to character the pathways that were actively regulated by miRNAs in the mammary gland. In the present study, pathway analysis of miRNA targets revealed that Wnt, TGF-β, MAPK, Notch, and JAK-STAT signaling pathways may play key roles in the mammary gland in the process of heat stress. Wnt, EGFR, TGF-β, and insulin signaling pathways are known to play a key role in normal development of the mammary gland [44–47]. The MAPK pathway is an important regulator of mammary epithelial cell differentiation and function [48]. These reports are supported by our findings. In addition, MAPK signaling, PI3k-Akt signaling, and immune-regulatory are greatly influenced by miRNA-mediated regulation in Frieswal cattle [15]. The function analysis of differentially expressed miRNAs and their target genes suggested the effects of heat stress on signaling mechanisms. Although many target gene candidates were predicted by bioinformatics methods, structural verification and signaling pathways analysis in vitro need to be further performed to validate the relationship between miRNAs and mRNA.

## Conclusions

In this study, we characterized miRNAs expressed in dairy cattle mammary gland under heat stress and identified 483 known bovine miRNAs and 139 novel miRNAs, and the heat-dependent differential modulation of miRNAs. The results showed that significant enrichment of predicted target genes of differentially expressed miRNAs in several biological processes, including developmental process, cellular process, biological regulation, cell death, focal adhesion, and biosynthesis of secondary metabolites. Moreover, our data provides the valuable information of the role of miRNAs in heat response and may be helpful for developing miRNA-based biomarkers for the control of heat stress in cows. We might reduce heat stress damage of Holstein cows by up-regulating or down-regulating these differentially expressed miRNAs.

## Additional files

Additional file 1: The primers of real-time quantitative RT-PCR used in this study. (XLS 31 kb)

Additional file 2: Novel miRNAs identified in this study. (XLS 45 kb)

Additional file 3: The differential expression of miRNAs in heat stressed and non-heat stressed groups. (XLS 33 kb)

Additional file 4: *P*-values for the differentially expressed miRNAs. (XLS 23 kb)

## Abbreviations

miR, miRNA: MicroRNA
mRNA: Messenger RNA
